# The Effects of Propolis on Viral Respiratory Diseases

**DOI:** 10.3390/molecules28010359

**Published:** 2023-01-01

**Authors:** Marcin Ożarowski, Tomasz M. Karpiński

**Affiliations:** 1 Department of Biotechnology, Institute of Natural Fibres and Medicinal Plants—National Research Institute, Wojska Polskiego 71b, 60-630 Poznań, Poland; 2 Chair and Department of Medical Microbiology, Poznań University of Medical Sciences, Rokietnicka 10, 60-806 Poznań, Poland

**Keywords:** propolis, respiratory tract diseases, acute respiratory syndrome coronavirus, influenza viruses, parainfluenza virus, rhinoviruses, in vitro, in vivo, clinical trial

## Abstract

Propolis remains an interesting source of natural chemical compounds that show, among others, antibacterial, antifungal, antiviral, antioxidative and anti-inflammatory activities. Due to the growing incidence of respiratory tract infections caused by various pathogenic viruses, complementary methods of prevention and therapy supporting pharmacotherapy are constantly being sought out. The properties of propolis may be important in the prevention and treatment of respiratory tract diseases caused by viruses such as severe acute respiratory syndrome coronavirus 2, influenza viruses, the parainfluenza virus and rhinoviruses. One of the main challenges in recent years has been severe acute respiratory syndrome coronavirus 2 (SARS-CoV-2), causing COVID-19. Recently, an increasing number of studies are focusing on the activity of various propolis preparations against SARS-CoV-2 as an adjuvant treatment for this infection. Propolis has shown a few key mechanisms of anti-SARS-CoV-2 action such as: the inhibition of the interaction of the S1 spike protein and ACE-2 protein; decreasing the replication of viruses by diminishing the synthesis of RNA transcripts in cells; decreasing the particles of coronaviruses. The anti-viral effect is observed not only with extracts but also with the single biologically active compounds found in propolis (e.g., apigenin, caffeic acid, chrysin, kaempferol, quercetin). Moreover, propolis is effective in the treatment of hyperglycemia, which increases the risk of SARS-CoV-2 infections. The aim of the literature review was to summarize recent studies from the PubMed database evaluating the antiviral activity of propolis extracts in terms of prevention and the therapy of respiratory tract diseases (in vitro, in vivo, clinical trials). Based upon this review, it was found that in recent years studies have focused mainly on the assessment of the effectiveness of propolis and its chemical components against COVID-19. Propolis exerts wide-spectrum antimicrobial activities; thus, propolis extracts can be an effective option in the prevention and treatment of co-infections associated with diseases of the respiratory tract.

## 1. Introduction

Global epidemiological challenges have resulted in natural substances becoming increasingly important due to their wide availability and popularity. The increasing resistance to many anti-viral drugs and their low efficacy against viral diseases make it necessary to analyze the results of studies carried out to ascertain the therapeutic effects of natural substances such as propolis. Propolis has been used for medical purposes since ancient times. Propolis in various forms is a natural product that is widely accepted by patients around the world in various health situations. In traditional folk medicine, propolis has been used in the treatment of various diseases, particularly for anti-inflammatory, anti-bacterial, anti-fungal and anti-ulcer purposes. Moreover, propolis has long been used to improve health and prevent many diseases [1,2]. A number of studies demonstrated the popularity of propolis in the treatment of respiratory tract infections [3,4], alone or in combination with another immunomodulator such as *Echinacea* sp. [5].

Infectious diseases involving viral and bacterial respiratory pathogenic microorganisms have been shown to significantly impact lives and impart economic costs [6]. According to Ferkol et al. [7], it was observed that respiratory tract diseases such as infections of the respiratory tract, influenza and asthma caused the highest societal and economic burdens. One of the main challenges in recent years has been severe acute respiratory syndrome coronavirus 2 (SARS-CoV-2), causing COVID-19, with a calculated direct medical cost of USD 163.4 billion [8]. Therefore, there is a need to search for cheaper and more effective methods for the treatment of respiratory diseases. So far, the treatment of patients with COVID-19 is based on the use of many drugs, including antivirals, anti-inflammatory drugs, antibodies obtained from convalescent plasma, anticoagulants and monoclonal antibodies [9]. Pharmacological studies have demonstrated that propolis may exert synergistic effects when used with various groups of medicinal products such as antibiotics, antifungals and antiviral drugs. Furthermore, it is believed that propolis can lead to a reduction in the required doses of these drugs [10]. In addition to propolis, over 30 medicinal plant species used in the therapy of SARS-CoV-2 infection have been described (e.g., *Forsythia suspensa* (Thunb.) Vahl, *Glycyrrhiza glabra* L., *Platycodon grandiflorum* (Jacq.) A. DC., *Nigella sativa* L.) [11,12,13,14,15].

Recently, there has been an increase in the number of studies examining the various propolis preparation methods (extracts, liposomes) and their efficacy against severe acute respiratory syndrome coronavirus 2 (SARS-CoV-2) as an adjuvant treatment for this infection [6,16,17,18,19,20,21,22,23,24,25,26,27,28,29]. Therefore, this review aims to provide a summary of recent preclinical (in vitro and in vivo) and clinical studies on the role of propolis and its extracts in the prevention and treatment of viral respiratory diseases.

## 2. Methodology

We searched the PubMed database for scientific articles using the following search terms (keywords): “propolis”, “respiratory diseases”, “COVID-19”, “influenza viruses”, “parainfluenza virus”, “rhinoviruses”. We also collected information on clinical trials from the platform clinical.trial.gov.

In the PubMed database, 4259 publications about “propolis” are available; however only 228 publications are about the antiviral activity of propolis, including 130 articles from the last 10 years (2012–2022). In fact, publications relating to the use of “propolis” in “respiratory diseases” numbered only 25 between the years of 1976 to 2022 and only 18 between 2012 and 2022. With respect to the antiviral activity of “propolis” against “COVID-19”, the PubMed database contains 57 scientific papers published over a two-year period (from 2020 up to 2022). Specifically, 16 articles were published in 2020, 24 in 2021 and 17 in 2022. Most of these are review articles and studies using in vitro and animal models, with only a few studies (*n* = 8) having been carried out by in silico means. To date, the results of only three clinical studies are available in this database and one on the platform clinical.trial.gov. Moreover, with respect to the antiviral activity of propolis against influenza viruses, PubMed contains 11 publications, while only 2 publications relate to the effect of propolis on rhinoviruses. In short, we included publications from the last 10 years, but where it was necessary (in the absence of current research), previously published articles were cited. The discussion features publications pertaining to respiratory diseases. It should likewise be emphasized that the article by Serkedjiev et al., published in 1992, is not available in PubMed; however, it is often cited by other authors and as such has been included in the present review.

## 3. Progress in Studies of Propolis

**Figure 1:**
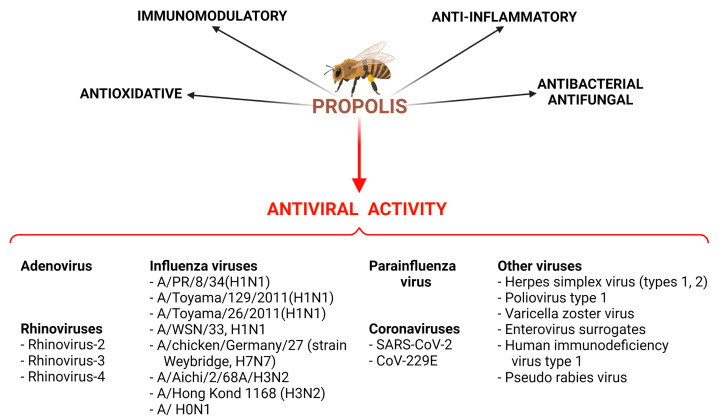
Activities of propolis extracts and their natural chemical compounds demonstrated during in vitro and in vivo studies. Created with BioRender.com.

Many studies examined the anti-microbial activities of propolis, including not only antibacterial and antifungal properties [30,31,32] but also its antiviral activity. According to Yosri et al. [33], ethanolic extracts of propolis have anti-viral properties against the Herpes simplex virus (types 1 and 2) in vitro; however, only six such in vitro studies have been carried out. Moreover, extracts of propolis can exert activity against Poliovirus type 1, but the results of only two in vitro studies have been described. Yosri et al. [33] summarized the activity of propolis against the Varicella zoster virus (one study), Enterovirus surrogates (one study), the Influenza A virus H1N1 (two studies), human immunodeficiency virus type 1 (one study) and Pseudo Rabies Virus (one study). According to Münstedt [34], six randomized trials have been conducted in order to estimate the anti-viral activity of propolis; it was found that propolis was superior to a placebo against herpes simplex virus type 1, herpes simplex virus type 2 and the varicella zoster virus (Figure 1).

**Figure 2:**
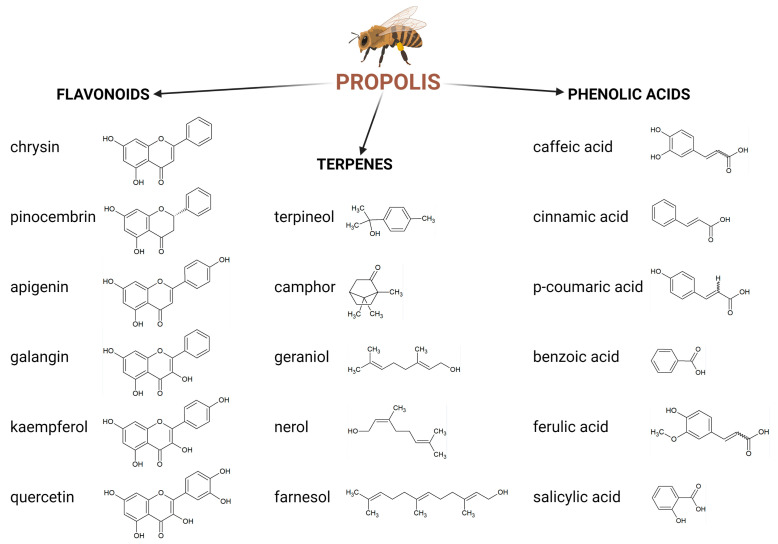
Main chemical compounds of propolis. Created with BioRender.com.

More studies have been conducted for single chemical compounds found in propolis and other plants. Anti-viral activity has been observed with phenolic compounds such as apigenin and kaempferol (against various types of the Influenza A virus) [35], acacetin, caffeic acid, chrysin, ferulic acid, fisetin, luteolin, p-coumaric acid and quercetin (against various types of the human rhinovirus) [36]. Progress has been made with regard to identifying the chemical compounds responsible for the pharmacologic and therapeutic effects found in propolis extracts (Figure 2). Ethanolic extracts from propolis may be of more interest because they contain many chemical compounds that can exert synergistic activity against various types of viruses. Several papers described that extracts from natural products are a source of complex matrices of bioactive metabolites, showing activity through synergy [31,32,37,38,39]. To date, over 800 chemical compounds have been detected in various types of propolis from different geographical areas [39]. Zullkiflee et al. [40] describe the main chemical composition of propolis and highlight compounds such as aromatic acids, alcohols, esters, fatty and aliphatic acids, flavonoids, microelements, sugars, vitamins and others.

An increasing number of studies are focusing on the efficacy of using various propolis preparations (extracts, liposomes) against SARS-CoV-2 as an adjuvant treatment for this infection [16,17,18,19,20,21,22,23,24,25,26,27,28,29,40].

## 4. Propolis Activity against Coronaviruses

### 4.1. Preclinical Studies

**Figure 3:**
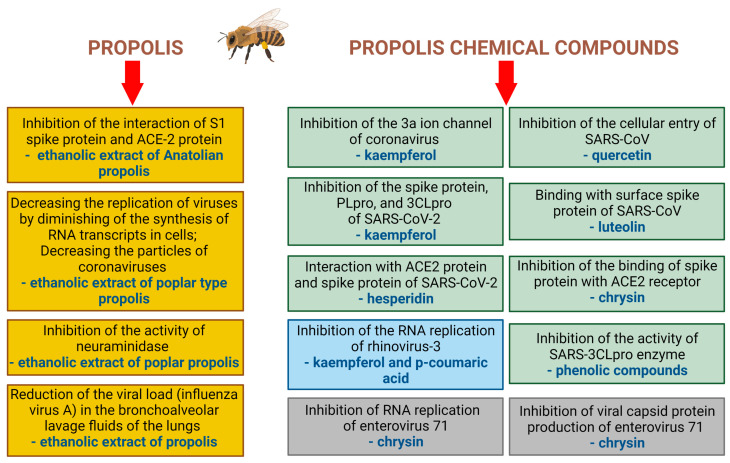
Examples of antiviral mechanisms of propolis and its chemical compounds (in vitro studies). Created with BioRender.com.

There is evidence suggesting that propolis extracts exhibit antiviral effects against the SARS-CoV-2 infection through mechanisms such as: inhibiting the interaction of the S1 spike protein and ACE-2 protein; decreasing the replication of viruses by diminishing the synthesis of RNA transcripts in cells; decreasing the number of virions, inhibiting the activity of neuraminidase; reducing the viral load (influenza virus A) in the bronchoalveolar lavage fluids of the lungs. These antiviral mechanisms of action of propolis and its chemical compounds (in vitro studies) have been summarized in Figure 3.

Moreover, it was shown that quercetin exhibited in vitro activity against human CoV-229E [41]. Ali et al. [42] highlighted that flavonoids such as naringin and rutin, which are found in propolis, showed concentration-dependent anti-SARS-CoV-2 activity in vitro. Yosri et al. [33], observed that such anti-viral effects are also exhibited by broussoflavonol F, chrysin, glyasperin A, kaempferol, sulabiroins A, caffeic acid, 3-phenyllactic acid, lumichrome. Moreover, various secondary metabolites exhibit antiviral action; specifically, phenolic compounds (inhibit the activity of SARS-3CLpro enzyme), quercetin (inhibits the cellular entry of SARS-CoV and the activity of SARS-CoV 3CLpro), kaempferol (inhibits the 3a ion channel of coronavirus), luteolin (binds the surface spike protein of SARS-CoV), apigenin and luteolin (inhibit the activity of SARS-CoV 3CLpro), [13,43,44,45]. Additionally, chrysin exhibited antiviral activity (via the inhibition of viral capsid protein production and the RNA replication of enterovirus 71) [46]. Recently, Jamshidnia et al. [15] reported that other naturally occurring chemical compounds (curcumin, kaempferol, licoleafol, myricitrin, silybin) inhibited the spike protein, PLpro and 3CLpro of SARS-CoV-2. Furthermore, Malekmohammad and Rafieian-Kopaei [14] found that not only quercetin, apigenin, kaempferol and curcumin, but also baicalin, glycyrrhizin, scutellarin and ursolic acid may be considered as candidates for the treatment of the symptoms linked with SARS-CoV-2 infection. Moreover, it was shown that chrysin inhibits the binding of the spike protein with the ACE2 receptor [47]. A molecular docking study [48] revealed that hesperidin can interact with the ACE2 protein and spike protein of SARS-CoV2.

Recently, Silva-Beltrán et al. [41] showed that extracts from green and brown propolis had anti-viral activity against human CoV-229E in MRC-5 cells (EC_50_ = 19.08 µg/mL and 11.24 µg/mL, respectively). Moreover, a number of studies outlined the anti-SARS-CoV-2 activity of propolis extracts in vitro [19,20,22,26]. Berretta et al. [26] reviewed the potential antiviral mechanisms of propolis and various natural chemical compounds against-SARS-CoV-2. The authors observed that, through interactions with angiotensin-converting enzyme 2 (ACE-2) and transmembrane serine protease 2 (TMPRSS2), propolis reduced the invasion of SARS-CoV-2 to the host cells. These enzymes are keys targets for effective treatment options because SARS-CoV-2 strongly binds to ACE-2, thus inducing pathogenetic pathways. However, only three in vitro studies relating to propolis have been performed in this field. The results of a study conducted by Güler et al. [22] showed that a 70% ethanolic extract (with a higher content of polyphenols) of Anatolian propolis inhibited the interaction of the S1 spike protein (SARS-CoV-2) and the ACE-2 protein. The study also employed an ELISA kit assay and in silico methods to show that hesperetin and pinocembrin exhibit inhibitory activity against the SARS-CoV-2 S1 spike protein and ACE-2 protein (IC_50_ = 11.13 mM and IC_50_ = 14.15 mM, respectively) [22]. Moreover, Sberna et al. [19] observed that an 80% extract of poplar-type propolis at a concentration of 25 µg/mL, added to a kidney epithelial cell line and human lung epithelial cells with SARS-CoV-2 infection, decreased viral replication and diminished the synthesis of RNA transcripts in cells. Moreover, propolis extract decreased the number of virions and caused a reduction in the number of infected cells [19].

Although several significant studies utilizing in vitro models have been performed, there is no systematic research satisfactorily comparing the activity of different types of propolis from different regions of the world, However, many review articles combine the multidirectional activity against various viruses, for both single active compounds and extracts of propolis (Figure 3). As such, the quantity and quality of research on the antiviral activity of propolis against SARS-CoV-2 are still unclear.

### 4.2. Clinical Trials

Based on the review of the literature, it was found that in recent years studies have focused mainly on the assessment of the effectiveness of propolis and its chemical components against COVID-19. However, with respect to using propolis as an adjunct therapy for patients with COVID-19, only three trials were carried out [48,49,50] and one clinical study is in progress (no. NCT04916821). Recently, most attention has been given to a randomized and open-label clinical study performed at the Hospital São Rafael in Brazil (NCT04480593) [4,16,20,26,42,48]. In this clinical study [48], two groups of patients with COVID-19 (from 18 to 80 years of age) received an extract of green propolis (Propomax^®^ capsules) at doses of 400 mg (40 patients) and 800 mg (42 patients) per day for 7 days, in addition to standard hospital therapy. The results and observations showed that the patients receiving propolis experienced a reduction in their hospitalization time.

Another randomized, double-blind and placebo-controlled clinical trial sought to determine the efficacy of an extract from Iranian green propolis in reducing clinical symptoms in patients with COVID-19 (from 18 to 75 years of age, *n* = 40) at the Al-Zahra Hospital in Isfahan, Iran. A total of 300 mg of extract of propolis in tablets was administered three times a day for 14 days. An improvement in the clinical symptoms of COVID-19 was observed in patients receiving the propolis treatment compared to those who received the standard treatment. However, only a short summary is available [49].

A third randomized clinical trial [50] focused on the assessment of the therapeutic effects of propolis dissolved in isopropanol together with methanolic extract of *Hyoscyamus niger* given to patients with COVID-19 (from 18 to 65 years of age, *n* = 50) at the Akhavan and Sepehri Clinics, Kashan University of Medical Sciences in Iran. The syrup, which contained 1.6 mg of the extract of *H. niger* and 450 mg of propolis, was administered at a dose of 10 mL three times/day for 6 days. The observations showed that dry cough, breath score, sore throat and chest pain were decreased in patients receiving the syrup when compared to those patients who did not receive the syrup. These results have statistical significance.

Although promising, the aforementioned studies have many limitations, such as a lack of information about the types of viruses inducing symptoms of COVID-19 in the patients. The dose selection and the duration of the intervention may need to be optimized as well. At present there is a need for further research and clinical observation in order to draw clear conclusions about the efficacy of propolis as it relates to improving the condition of patients with SARS-CoV-2 infection.

On the ClinicalTrials.gov platform, another randomized, open-label clinical study with parallel assignment (no. NCT04916821) at the Trabzon Kanuni Education and Research Hospital in Turkey is also registered [51]. Forty-five participants with confirmed COVID-19 will be included. One group will be treated with an aqueous propolis extract (food supplement product) at a dose of 2 mL (50 mg/mL) orally given 3 times/day for 7 days. The second group will receive 1 mL of oily propolis extract (64 mg/mL) with 1 mL oily perga extract (120 mg/mL) orally 3 times daily for 7 days. The study will examine the use of propolis extracts on various healing parameters in patients with COVID-19.

In addition to the above-mentioned clinical trials, two case reports have been described in the literature [21,52]. According to Fiorini et al. [21], one patient from Brazil (a 52-year-old woman) with symptoms of COVID-19 who received a solution of Brazilian green propolis (30%) at a dose of 45 drops, three times daily for 14 days, reported improvement in the severity of symptoms after 12 days. Moreover, one patient from Turkey (a 38-year-old man) with symptoms of COVID-19 received a solution of Anatolian propolis (30%) at a dose of 20–80 drops per day for 1 month together with other therapeutics such as hydroxychloroquine, favipiravir, tocilizumab. This method of intervention yielded an improvement of the clinical symptoms of the disease after 7 days [52]. Moreover, it should be highlighted that the effects of propolis alone or in combination with antiviral drugs must be studied not only using in vitro methods, but likewise through the use of clinical trials.

According to Yusuf [53], propolis as a source of phenolic compounds can be considered as one of the best therapeutic options for patients with COVID-19 and diabetes mellitus. This therapeutic solution is justified by the fact that hyperglycemia may increase the risk (severity?) of SARS-CoV-2 infections; propolis decreases the synergy between SARS-CoV-2 and COVID-19-mediated morbidity. Ghosh [17] highlights that patients with diagnosed COVID-19 often have one or more comorbidities (e.g., chronic lung disease, cardiovascular disease and hypertension). Balica et al. [54] reviewed 22 preclinical and 8 clinical studies and found that propolis use produced favorable outcomes in patients with diabetes mellitus, dyslipidemia and obesity.

Many studies revealed that propolis use exerted a beneficial effect in chronic obstructive pulmonary disease by decreasing the level of proinflammatory cytokines and reducing acute lung inflammation [40,55]. Moreover, propolis exerted anticoagulant, antihypertensive and anti-hemostatic effects [56]. It must be noted that propolis exerts wide-spectrum antimicrobial activities [4,31,32] against pathogens that may infect a patient during the pathogenesis of COVID-19 as co-infections and secondary bacterial infections. The mortality data on secondary bacterial infections are scarce (Farrell [57]). COVID-19 infection may lead to the damage of the lung parenchyma, with long-term complications such as bacterial infections being more likely in patients with the disease. According to Pourajam [58], secondary bacterial infection caused by *Klebsiella pneumoniae* and *Acinetobacter baumannii* was observed in 11.9% of patients. Others highlight that secondary infection *Chlamydia pneumonia, Haemophilus influenza, Legionella pneumophila, Mycoplasma pneumoniae, Streptococcus pneumoniae, Staphylococcus aureus* have also been observed [59]. Recently, it was found that propolis exhibits antibacterial activity not only against *Klebsiella pneumoniae* but also *Enterococcus faecalis, Listeria monocytogenes*, *Pseudomonas aeruginosa*, *Staphylococcus aureus*, *Streptococcus sobrinus*, *Streptococcus mutans* [60,61], *Bacillus subtilis, Clostridium diffcile, Escherichia coli, Proteus mirabilis, Salmonella spp.* and *Streptococcus epidermidis* [32].

## 5. Propolis Activity against Influenza A Virus and Parainfluenza Virus

### 5.1. Preclinical Studies

There are four types of influenza viruses (A–D). However, only subtypes of the influenza A and B viruses cause seasonal epidemics of disease [62]. According to the WHO [63], these annual epidemics are estimated to cause approximately 3 to 5 million cases of serious disease and 650,000 deaths resulting from respiratory tract complications.

A study performed by Governa et al. [64] focused on the assessment of an 80% ethanolic extract of poplar propolis (containing the flavonoids galagin and pinocembrin) against a subtype of the H1N1 virus in vitro. They found that this extract (35 μg/mL) inhibited the activity of neuraminidase (IC_50_, 35.29 µg/mL), an enzyme involved in the viral lifecycle. Poplar propolis extract likewise possessed anti-inflammatory and immunomodulatory activities.

A few years earlier, the water and the ethanolic extracts of Brazilian green propolis (200 mg/kg), 3,4-dicaffeoylquinic acid (3,4-diCQA, 50 mg/kg) and chlorogenic acid (50 mg/kg) were tested in Balb/c mice infected with the influenza A virus strain A/WSN/33 [65]. The investigators found that the propolis extract and 3,4-diCQA resulted in an increased survival rate in mice as well as the upregulation of the TNF-related apoptosis-inducing ligand; however, chlorogenic acid did not show these effects.

Urushisaki et al. [66] investigated the effects of Brazilian green propolis water extract on two influenza strains (A/WSN/33, H1N1) in vitro and found that this extract exerts cytoprotective effects. 3,4-dicaffeoylquinic acid (included in the extract) was the most active against the influenza viruses tested (EC_50_ = 81.1 μM) when compared with other active chemical compounds such as caffeoylquinic acids and caffeic acid. Chlorogenic acid showed the lowest antiviral activity and quinic acid was deemed ineffective.

Previously, it was shown that a 70% ethanolic extract of propolis exerted antiviral activity against the avian influenza virus A/chicken/Germany/27 (strain Weybridge, H7N7) in vitro [67]. Moreover, the ethanolic extract of Brazilian propolis at doses of 2 and 10 mg/kg administered three times daily to mice (DBA/2) infected by the influenza A/PR/8/34 virus prolonged the survival time of infected mice and improved the severity of influenza symptoms in these animals [68]. According to Serkedjieva et al. [69], it has been observed that the Et_2_O fraction of the ethanolic extract of propolis diminished the infectious activity of A/H1N1 and A/H3N2 in vitro at concentrations of 50 µg/mL and 100 µg/mL, respectively. Results cited by Serkedjieva et al. [69] suggest that propolis was also active against the H0N1 viral strain in vitro. Furthermore, kaempferol at a dose of 30 mg/kg prolonged the survival time of animals (BALF mice) after infection [35]. Moreover, apigenin, coumaric acid and kaempferol showed antiviral activity against strains such as A/PR/8/34(H1N1), A/Toyama/129/2011(H1N1), A/Toyama/26/2011(H1N1) in vitro when compared with other tested chemical compounds (i.e., artepillin C, chrysin, quercetin, rutin, benzoic acid, 4-hydroxy-3-methoxycinnamic acid, trans-cinnamic acid). However, caffeic acid exerted activity against the A/Toyama/129/2011(H1N1) and A/Toyama/26/2011(H1N1) strains but not the A/PR/8/34(H1N1) strain in vitro [35]. Additionally, Serkedjieva et al. [69] showed that some synthetic constituents of propolis decreased the infectious activity of the influenza viruses A/Hong Kong/1/68 (H3N2) and A/PR/8/34 (H1N1) in vitro. Drago et al. [70] compared the antiviral activity of the hydroalcoholic extract from propolis. They found that the antiviral activity of Actichelated^®^ propolis (in concentrations ranging from 0.032 to 0.128 g/l), in conjunction with galagin, was higher than that of the hydroalcoholic extract against the influenza virus, parainfluenza virus, adenovirus and herpes virus type 1. Propolis also possesses antibacterial activity and does not show cytotoxic effects. Table 1 contains summary of the antiviral activity of propolis against respiratory viruses obtained in preclinical studies.

### 5.2. Clinical Trials

To date, there have been no published controlled clinical trials focusing on the evaluation of the efficacy of propolis preparations against infections caused by influenza viruses.

## 6. Activity against Human Rhinoviruses

### 6.1. Preclinical Studies

Upper respiratory tract infections are among the most common diseases in the world [71]. Human rhinoviruses, coronaviruses and respiratory syncytial viruses are responsible for infections of the upper respiratory tract not only in children but also in adults. It is estimated that they cause more than 50% of common colds [36]. According to the estimation of Jin et al. [71], the incident cases of upper respiratory tract infections numbered at approximately 17.2 billion in 2019. Despite the great burden to health, recently only one in vitro study investigated the activity of propolis in respiratory tract infections. Kwon et al. [36] outline that an 80% ethanolic extract of Brazilian propolis and its fractions obtained using hexane, chloroform and ethyl acetate exhibit antiviral activity against rhinovirus-4 with IC_50_ values ranging from 5.00 µg/mL (chloroform-soluble fraction) to 15.4 µg/mL (ethanolic extract). Kaempferol and chrysin were obtained from this ethanolic extract. Kaempferol showed the most antiviral activity against rhinovirus-2, with IC_50_ values of 7.3 µg/mL, while chrysin showed the most antiviral activity against rhinovirus-3 (IC_50_ = 17.3 µg/mL). Other natural chemical compounds found to be active against rhinovirus-2 include quercetin, luteolin and galagin. Moreover, kaempferol and p-coumaric acid inhibited the viral RNA replication levels of rhinovirus-3 in HeLa cells and reduced the penetration of the viruses into the cells [36].

### 6.2. Clinical Trials

With respect to clinical trials, the authors highlight the use of bee products in the treatment of upper respiratory tract infections in children [3]. The results of a double-blind clinical trial involving young patients ranging from 5 to 12 years of age with viral and bacterial tonsillopharyngitis showed that the administration of a complex product containing honey, royal jelly and propolis (20–40 mg/kg for 10 days) was beneficial in the treatment of upper respiratory tract infections [3]. Recently, Esposito et al. [72] performed a randomized, double-blind placebo-controlled clinical trial in order to assess the efficacy of an oral propolis spray (M.E.D.^®^ propolis) in patients with symptoms of upper respiratory tract diseases (*n* = 58, from 18 to 77 years of age). Propolis was administered at a dose of 2–4 sprays (0.8–1.6 mL of propolis) three times per day (5 days). The remission of symptoms was observed in 83% of patients after three days of therapy. Previously, Di Pierro et al. [73] showed that a mixture of propolis-phytosome (Propolisina^®^) containing a 75 mg/sachet of pure propolis was effective in an open-label, retrospective, controlled clinical study involving patients with nonstreptococcal and viral pharyngitis caused by paramyxoviruses, rhinoviruses, adenoviruses. This product decreased the severity of symptoms such as sore throat, fever and pharyngeal erythema.

Additionally, Cohen et al. [74] drew attention to a herbal preparation containing propolis (50 mg/mL), Echinacea (50 mg/mL) and vitamin C (10 mg/mL) in a randomized, double-blind, placebo-controlled study in order to assess its effectiveness in preventing upper respiratory tract infections in children (from 1 to 5 years of age). This preparation was administered to patients at doses of 5.0 mL and 7.5 mL twice daily for 12 weeks. Patients receiving the preparation experienced a 55% reduction in the number of illness episodes and a 62% reduction in the number of days with fever, as well as an overall decrease in the total number of days with symptomatic illness. According to Salatino et al. [10], the number of clinical studies has increased; however, most were carried out with the herpes simplex and influenza viruses. In fact, recent review articles are still describing old data on the antiviral activities of propolis. Table 2 contains summary of antiviral activity of propolis against viral respiratory diseases based on clinical studies.

## 7. Conclusions

There are many mechanisms by which propolis exerts its antimicrobial effects, with more continuously being elucidated. Many articles outline a wide range of actions of propolis against microorganisms. Experimental studies (in vitro and in vivo) as well as clinical trials have shown that propolis extracts are an effective option in the prevention and treatment of respiratory tract diseases caused by viruses such as severe acute respiratory syndrome coronavirus 2, influenza viruses, the parainfluenza virus and rhinoviruses. Propolis likewise shows anti-inflammatory activity, thus offering a potential mechanism to combat the cytokine storm observed during some SARS-CoV-2 infections. Despite recent advances in the field, the authors suggest that more research needs to be undertaken to establish the effects of combining propolis with conventional antiviral medications. Such a combination is promising because of the potential synergistic effects between propolis, its biologically active compounds and existing antiviral medications. Furthermore, propolis has been shown to be beneficial in the management of hyperglycemia and hypertension; thus, extracts can be used to lessen the burden imposed by comorbid diseases in patients with respiratory tract infections. To the best of the authors’ knowledge, there is no systematic research being conducted on the activity of different types of propolis with respect to the treatment of respiratory tract diseases at the time of writing. Moreover, it is evident that despite the growing body of knowledge with respect to the mechanisms of action of propolis, there is a pronounced lack of clinical trials involving its use.

## Figures and Tables

**Table 1:** Summary of the antiviral activity of propolis against respiratory viruses obtained in *n* preclinical studies.

Type of Virus	Type of Preparation of Propolis	Effects	Types of Studies	Ref.
CoV-229E	Extracts from green and brown propolis	Anti-viral activity against human CoV-229E after application of green propolis extract (EC_50_ = 19.08 µg/mL) and brown propolis extract (EC_50_ = 11.24 µg/mL)	In vitro: line of MRC-5 cells	Silva-Beltrán et al. [41]
SARS-CoV-2	70% ethanolic extract of Anatolian propolis	Inhibition of the interaction between the SARS-CoV-2 S1 spike protein and ACE-2 receptors in a concentration- dependent manner	In vitro: screening colorimetric assay kit	Güler et al. [22]
SARS-CoV-2	80% extract of poplar-type propolis	Decrease in the replication of viruses by diminishing of the synthesis of RNA transcripts in cells after application of extract at a concentration of 25 µg/mL Decrease in the number of virions and a reduction in the number of infected cells	In vitro: VERO E6 (African green monkey, kidney epithelial cell line), CALU3 (human lung epithelial cell lines)	Sberna et al. [19]
Avian influenza virus A/chicken/Germany/27 (strain Weybridge, H7N7)	70% ethanolic extract from samples of propolis from Albania, Brazil, Bulgaria, Mongolia	Antiviral activity in eight samples of propolis Significant selectivity index (SI) for samples of Bulgarian propolis (SI = 8) and Brazilian propolis (IS = 35)	In vitro: primary chick embryo fibroblast (CEF) cells	Kujumgiev, 1999 [67]
A/H1N1 and A/H3N2	Et_2_O fraction of ethanolic extract of propolis	Decreasing the infectious activity of A/H1N1 and A/H3N2 in vitro at concentrations of 50 µg/mL and 100 µg/mL, respectively	In vitro: embryonated hen’s eggs	Serkedjieva, 1992 [69]
Influenza A/PR/8/34 virus	Ethanolic extract of Brazilian propolis	Increased survival time of infected mice and improved the symptoms of influenza in animals after 10 mg/kg administered three times daily Values of EC_50_ were from <10 to 116.6 µg/mL for thirteen samples	In vivo: mice (DBA/2) infected by influenza virus In vitro: Madin–Darby canine kidney (MDCK) cells	Shimizu, 2008 [68]
Influenza virus, parainfluenza virus, adenovirus	Actichelated^®^ propolis; hydroalcoholic extract from propolis	Antiviral activity of Actichelated^®^ propolis at concentrations from 0.032 g/l to 0.128 g/l is higher than that of hydroalcoholic extract against influenza virus, parainfluenza virus, adenovirus; no cytotoxic effects	In vitro: Hep-2 cell monolayer with isolated viruses	Drago, 2007 [70]
Influenza virus A/WSN/33 (H1N1)	Water extract of Brazilian green propolis	Increased cell viability at concentrations of 100 to 300 μg/mL of propolis extract Cell survival EC50 = 183 μg/mL	In vitro: Madin–Darby canine kidney (MDCK) cells	Urushisaki, 2011 [66]
Influenza A virus (H1N1)	80% ethanolic extract of Italian propolis	Inhibition of viral replication Inhibiting the neuraminidase activity IC50 = 35.29 µg/mL	In vitro: Madin-Darby canine kidney (MDCK) cells	Governa, 2019 [64]
Human rhinovirus: HRV-2, HRV-3, HRV-4	80% ethanolic extract of Brazilian propolis and its fractions obtained using hexane, chloroform and ethyl acetate, butanol, water	antiviral activity against HVR-4 with the following IC_50_ values: 5.00 µg/mL-chloroform-soluble fraction 5.2 µg/mL-ethyl acetate-soluble fraction 8.9 µg/mL-hexane-soluble fraction 15.4 µg/mL-ethanolic extract 26.7 µg/mL-water-soluble fraction 78.4 µg/mL-butanol-soluble fraction	In vitro: human epithelial adenocarcinoma cervix cell line HeLa (ATCC CCL-2)	Kwon, 2019 [36]

**Table 2:** Summary of antiviral activity of propolis against viral respiratory diseases based on clinical studies.

Type of Virus/Disease	Type of Preparation of Propolis; Dosage and Method of Administration	Population	Effects	Type of Study	Ref.
SARS-CoV-2	Standardized extract of green propolis (Propomax^®^ capsules) Extract of propolis at doses of 400 mg (40 patients) and 800 mg (42 patients) per day for 7 days	Patients with COVID-19 from 18 to 80 years of age, *n* = 82	Reduction in their hospitalization time; improvement in clinical symptoms of COVID-19	Pilot randomized clinical study	Silveira et al. [48]
SARS-CoV-2	Extract of Iranian green propolis 300 mg of propolis extract in tablets administered three times a day for 14 days	Patients with COVID-19 from 18 to 75 years of age, *n* = 40	Improvement in clinical symptoms of COVID-19	Randomized, double-blind, placebo-controlled clinical trial	Miryan et al. [49]
SARS-CoV-2	Propolis with extract of *Hyoscyamus niger* Syrup at dose of 10 mL three times a day for 6 days	Patients with COVID-19 from 18 to 65 years of age, *n* = 50	Decrease in dry cough, breath score, sore throat and chest pain	Randomized clinical study	Kosari et al. [50]
SARS-CoV-2	Aqueous propolis extract at a dose of 2 mL (50 mg/mL) orally given 3 times a day for 7 days, or at a dose of 1 mL (64 mg/mL) with 1 mL oily perga extract (120 mg/mL) given orally 3 times daily for 7 days	Patients with COVID-19	In progress	Randomized clinical study	NCT04916821 [51]
Mixed etiology: acute otitis media and/or nonstreptococcal pharyngitis	Mixture of propolis-phytosome (Propolisina^®^) containing 75 mg/sachet of pure propolis	Children over 2 years of age, *n* = 28	Effective in patients with nonstreptococcal and viral pharyngitis caused by paramyxoviruses, rhinoviruses, adenoviruses. Propolis decreased symptoms such as sore throat, fever and pharyngeal erythema	Open-label, retrospective, controlled clinical study	Di Pierro et al. [73]
Mixed etiology of upper respiratory tract diseases	Herbal preparation of propolis (50 mg/mL), Echinacea (50 mg/mL) and vitamin C (10 mg/mL) at doses of 5.0 mL and 7.5 mL twice daily for 12 weeks	Children from 1 to 5 years of age	55% reduction in the number of illness episodes, 62% reduction in the number of days with fever and decrease in the total number of days with symptoms of respiratory illness	Randomized, double-blind, placebo-controlled study	Cohen et al. [74]
Mixed etiology of upper respiratory tract diseases	Oral spray of propolis (M.E.D.^®^ propolis) at a dose of 2–4 sprays (0.8–1.6 mL of propolis) three times per day (5 days)	Patients from 18 to 77 years of age, *n* = 58	Remission of symptoms after three days of medication (in 83% of patients)	Randomized, double-blind, placebo-controlled clinical trial	Esposito et al. [72]
Mixed etiology: viral and bacterial tonsillopharyngitis	Complex product containing honey, royal jelly and propolis at a dose of 20–40 mg/kg for 10 days	Patients from 5 to 12 years of age	Effective in the treatment of infections of the upper respiratory tract	Double-blind clinical trial	Seçilmiş, 2020 [3]

## Data Availability

Not applicable.

## References

[1] Kuropatnicki A.K., Szliszka E., Krol W. (2013). Historical aspects of propolis research in modern times. Evid. Based Complement. Altern. Med..

[2] Rojczyk E., Klama-Baryła A., Łabuś W., Wilemska-Kucharzewska K., Kucharzewski M. (2020). Historical and modern research on propolis and its application in wound healing and other fields of medicine and contributions by Polish studies. J. Ethnopharmacol..

[3] Seçilmiş Y., Silici S. (2020). Bee product efficacy in children with upper respiratory tract infections. Turk J. Pediatr..

[4] Magnavacca A., Sangiovanni E., Racagni G., Dell'Agli M. (2022). The antiviral and immunomodulatory activities of propolis: An update and future perspectives for respiratory diseases. Med. Res. Rev..

[5] Yuksel S., Akyol S. (2016). The consumption of propolis and royal jelly in preventing upper respiratory tract infections and as dietary supplementation in children. J. Intercult. Ethnopharmacol..

[6] Zulhendri F., Perera C.O., Tandean S., Abdulah R., Herman H., Christoper A., Chandrasekaran K., Putra A., Lesmana R. (2022). The Potential use of propolis as a primary or an adjunctive therapy in respiratory tract-related diseases and disorders: A systematic scoping review. Biomed. Pharmacother..

[7] Ferkol T., Schraufnagel D. (2014). The global burden of respiratory disease. Ann. Am. Thorac. Soc..

[8] Richards F., Kodjamanova P., Chen X., Li N., Atanasov P., Bennetts L., Patterson B.J., Yektashenas B., Mesa-Frias M., Tronczynski K. (2022). Economic burden of COVID-19: A Systematic Review. ClinicoEcon. Outcomes Res..

[9] Ripari N., Sartori A.A., Honorio M.S., Conte F.L., Tasca K.I., Santiago K.B., Sforcin J.M. (2021). Propolis antiviral and immunomodulatory activity: A review and perspectives for COVID-19 treatment. J. Pharm. Pharmacol..

[10] Salatino A. (2022). Perspectives for Uses of Propolis in Therapy against Infectious Diseases. Molecules.

[11] Yeh C.F., Wang K.C., Chiang L.C., Shieh D.E., Yen M.H., San Chang J. (2013). Water extract of licorice had anti-viral activity against human respiratory syncytial virus in human respiratory tract cell lines. J. Ethnopharmacol..

[12] Zhou W., Yin A., Shan J., Wang S., Cai B., Di L. (2017). Study on the rationality for antiviral activity of Flos Lonicerae Japonicae-Fructus Forsythiae herb couple preparations improved by chito-oligosaccharide via integral pharmacokinetics. Molecules.

[13] Khan M., Saddique M.A.B., Tahir H., Amjad M.D., Ahmad A., Masood U., Khan D. (2022). A Short Review on Key Role of Plants and their Extracts in Boosting up Immune Response to Combat COVID-19. Infect. Disord. Drug. Targets.

[14] Malekmohammad K., Rafieian-Kopaei M. (2021). Mechanistic aspects of medicinal plants and secondary metabolites against severe acute respiratory syndrome coronavirus 2 (SARS-CoV-2). Curr. Pharm. Des..

[15] Jamshidnia M., Sewell R.D.E., Rafieian-Kopaei M. (2022). An Update on Promising Agents against COVID-19: Secondary metabolites and mechanistic aspects. Curr. Pharm. Des..

[16] Dilokthornsakul W., Kosiyaporn R., Wuttipongwaragon R., Dilokthornsakul P. (2022). Potential effects of propolis and honey in COVID-19 prevention and treatment: A systematic review of in silico and clinical studies. J. Integr. Med..

[17] Ghosh S., Al-Sharify Z.T., Maleka M.F., Onyeaka H., Maleke M., Maolloum A., Godoy L., Meskini M., Rami M.R., Ahmadi S. (2022). Propolis efficacy on SARS-COV viruses: A review on antimicrobial activities and molecular simulations. Environ. Sci. Pollut. Res. Int..

[18] Soleymani S., Naghizadeh A., Karimi M., Zarei A., Mardi R., Kordafshari G., Esmaealzadeh N., Zargaran A. (2022). COVID-19: General strategies for herbal therapies. J. Evid. Based Integr. Med..

[19] Sberna G., Biagi M., Marafini G., Nardacci R., Biava M., Colavita F., Piselli P., Miraldi E., D’Offizi G., Capobianchi M.R. (2022). In vitro evaluation of antiviral efficacy of a standardized hydroalcoholic extract of poplar type propolis against SARS-CoV-2. Front Microbiol..

[20] Bijelic K., Hitl M., Kladar N. (2022). Phytochemicals in the Prevention and Treatment of SARS-CoV-2—Clinical Evidence. Antibiotics.

[21] Fiorini A.C., Scorza C.A., de Almeida AC G., Fonseca M., Finsterer J., Fonseca F.L., Scorza F.A. (2021). Antiviral activity of Brazilian green propolis extract against SARS-CoV-2 (severe acute respiratory syndrome-coronavirus 2) infection: Case report and review. Clinics.

[22] Güler H.İ., Ay Şal F., Can Z., Kara Y., Yildiz O., Beldüz A.O., Canakci S., Kolayli S. (2021). Targeting CoV-2 spike RBD and ACE-2 interaction with flavonoids of Anatolian propolis by in silico and in vitro studies in terms of possible COVID-19 therapeutics. Turk J. Biol..

[23] Harisna A.H., Nurdiansyah R., Syaifie P.H., Nugroho D.W., Saputro K.E., Prakoso F.C.D., Rochman N.T., Maulana N.N., Noviyanto A., Mardliyati E. (2021). In silico investigation of potential inhibitors to main protease and spike protein of SARS-CoV-2 in propolis. Biochem. Biophys. Rep..

[24] Lima W.G., Brito J.C.M., da Cruz Nizer W.S. (2020). Bee products as a source of promising therapeutic and chemoprophylaxis strategies against COVID-19 (SARS-CoV-2). Phyther. Res..

[25] Refaat H., Mady F.M., Sarhan H.A., Rateb H.S., Alaaeldin E. (2021). Optimization and evaluation of propolis liposomes as a promising therapeutic approach for COVID-19. Int. J. Pharm..

[26] Berretta A.A., Silveira M.A.D., Cóndor Capcha J.M., De Jong D. (2020). Propolis and its potential against SARS-CoV-2 infection mechanisms and COVID-19 disease: Running title: Propolis against SARS-CoV-2 infection and COVID-19. Biomed. Pharmacother..

[27] Bachevski D., Damevska K., Simeonovski V., Dimova M. (2020). Back to the basics: Propolis and COVID-19. Dermatol. Ther..

[28] Maruta H., He H. (2020). PAK1-blockers: Potential Therapeutics against COVID-19. Med. Drug Discov..

[29] Zulhendri F., Chandrasekaran K., Kowacz M., Ravalia M., Kripal K., Fearnley J., Perera C.O. (2021). Antiviral, antibacterial, antifungal, and antiparasitic properties of propolis: A review. Foods.

[30] Wieczorek P.P., Hudz N., Yezerska O., Horcinová-Sedlácková V., Shanaida M., Korytniuk O., Jasicka-Misiak I. (2022). Chemical Variability and Pharmacological Potential of Propolis as a Source for the Development of New Pharmaceutical Products. Molecules.

[31] Ozarowski M., Karpinski T.M., Alam R., Łochynska M. (2022). Antifungal Properties of Chemically Defined Propolis from Various Geographical Regions. Microorganisms.

[32] Przybyłek I., Karpiński T.M. (2019). Antibacterial Properties of Propolis. Molecules.

[33] Yosri N., Abd El-Wahed A.A., Ghonaim R., Khattab O.M., Sabry A., Ibrahim M.A.A., Moustafa M.F., Guo Z., Zou X., Algethami A.F.M. (2021). Anti-viral and immunomodulatory properties of propolis: Chemical diversity, pharmacological properties, preclinical and clinical applications, and in silico potential against SARS-CoV-2. Foods.

[34] Münstedt K. (2019). Bee products and the treatment of blister-like lesions around the mouth, skin and genitalia caused by herpes viruses -A systematic review. Complement. Ther. Med..

[35] Kai H., Obuchi M., Yoshida H., Watanabe W., Tsutsumi S., Park Y.K., Matsuno K., Yasukawa K., Kurokawa M. (2014). In vitro and in vivo anti-influenza virus activities of flavonoids and related compounds as components of Brazilian propolis (AF-08). J. Funct. Foods.

[36] Kwon M.J., Shin H.M., Perumalsamy H., Wang X., Ahn Y.J. (2020). Antiviral effects and possible mechanisms of action of constituents from Brazilian propolis and related compounds. J. Apic. Res..

[37] Sokolonski A.R., Fonseca M.S., Machado B.A.S., Deegan K.R., Araújo R.P.C., Umsza-Guez M.A., Meyer R., Portela R.W. (2021). Activity of antifungal drugs and Brazilian red and green propolis extracted with different methodologies against oral isolates of Candida spp.. BMC Complement. Med. Ther..

[38] Stähli A., Schröter H., Bullitta S., Serralutzu F., Dore A., Nietzsche S., Milia E., Sculean A., Eick S. (2021). In vitro activity of propolis on oral microorganisms and biofilms. Antibiotics.

[39] Kasote D., Bankova V., Viljoen A.M. (2022). Propolis: Chemical diversity and challenges in quality control. Phytochem. Rev..

[40] Zullkiflee N., Taha H., Usman A. (2022). Propolis: Its Role and Efficacy in Human Health and Diseases. Molecules.

[41] Silva-Beltrán N.P., Galvéz-Ruíz J.C., Ikner L.A., Umsza-Guez M.A., de Paula Castro T.L., Gerba C.P. (2022). In vitro antiviral effect of Mexican and Brazilian propolis and phenolic compounds against human coronavirus 229E. Int. J. Environ. Health Res..

[42] Ali A.M., Kunugi H. (2021). Propolis, bee honey, and their components protect against coronavirus disease 2019 (COVID-19): A review of in silico, in vitro, and clinical studies. Molecules.

[43] Schwarz S., Sauter D., Wang K., Zhang R., Sun B., Karioti A., Bilia A.R., Efferth T., Schwarz W. (2014). Kaempferol derivatives as antiviral drugs against the 3a channel protein of coronavirus. Planta Med..

[44] Chen C.J., Michaelis M., Hsu H.K., Tsai C.C., Yang K.D., Wu Y.C., Cinatl J., Doerr H.W. (2008). Toona sinensis Roem tender leaf extract inhibits SARS coronavirus replication. J. Ethnopharmacol..

[45] Yi L., Li Z., Yuan K., Qu X., Chen J., Wang G., Zhang H., Luo H., Zhu L., Jiang P. (2004). Small molecules blocking the entry of severe acute respiratory syndrome coronavirus into host cells. J. Virol..

[46] Wang J., Ting Z., Jiang D., Sheng C., Fan Y., Qi J. (2014). Anti-enterovirus 71 effects of chrysin and its phosphate ester. PLoS ONE.

[47] Basu A., Sarkar A., Maulik U. (2020). Molecular docking study of potential phytochemicals and their efects on the complex of SARS-CoV2 spike protein and human ACE2. Sci. Rep..

[48] Silveira M.A.D., De Jong D., Berretta A.A., Galvão E.B.D.S., Ribeiro J.C., Cerqueira-Silva T., Amorim T.C., da Conceicao L.F.M.R., Gomes M.M.D., Teixeira M.B. (2021). Efficacy of Brazilian green propolis (EPP-AF^®^) as an adjunct treatment for hospitalized COVID-19 patients: A randomized, controlled clinical trial. Biomed Pharmacother..

[49] Miryan M., Soleimani D., Dehghani L., Sohrabi K., Khorvash F., Bagherniya M., Sayedi S.M., Askari G. (2020). The effect of propolis supplementation on clinical symptoms in patients with coronavirus (COVID-19): A structured summary of a study protocol for a randomised controlled trial. Trials.

[50] Kosari M., Noureddini M., Khamechi S.P., Najafi A., Ghaderi A., Sehat M., Banafshe H.R. (2021). The effect of propolis plus Hyoscyamus niger L. methanolic extract on clinical symptoms in patients with acute respiratory syndrome suspected to COVID-19: A clinical trial. Phytother Res..

[51] Investigation of Clinical Effectiveness of Propolis Extracts as Food Supplements in Patients with SARS-CoV-2(COVID-19). Identifier: NCT04916821. NCT04916821.

[52] Zorlu D. (2021). COVID-19 and anatolian propolis: A case report. Acta Med. Mediterr..

[53] Yusuf A.P., Zhang J.Y., Li J.Q., Muhammad A., Abubakar M.B. (2022). Herbal medications and natural products for patients with covid-19 and diabetes mellitus: Potentials and challenges. Phytomed Plus.

[54] Balica G., Vostinaru O., Stefanescu C., Mogosan C., Iaru I., Cristina A., Pop C.E. (2021). Potential Role of Propolis in the Prevention and Treatment of Metabolic Diseases. Plants.

[55] Khayyal M.T., El-Ghazaly M.A., El-Khatib A.S., Hatem A.M., de Vries P.J., El-Shafei S., Khattab M.M. (2003). A clinical pharmacological study of the potential beneficial effects of a propolis food product as an adjuvant in asthmatic patients. Fundam. Clin. Pharmacol..

[56] Silva H., Francisco R., Saraiva A., Francisco S., Carrascosa C., Raposo A. (2021). The cardiovascular therapeutic potential of propolis—A comprehensive review. Biology.

[57] Farrell J.M., Zhao C.Y., Tarquinio K.M., Brown S.P. (2021). Causes and consequences of COVID-19-associated bacterial infections. Front. Microbiol..

[58] Pourajam S., Kalantari E., Talebzadeh H., Mellali H., Sami R., Soltaninejad F., Amra B., Sajadi M., Alenaseri M., Kalantari F. (2022). Secondary bacterial infection and clinical characteristics in patients with COVID-19 admitted to two intensive care units of an academic hospital in Iran during the first wave of the pandemic. Front. Cell. Infect. Microbiol..

[59] Hoque M.N., Akter S., Mishu I.D., Islam M.R., Rahman M.S., Akhter M., Islam I., Hasan M.M., Rahaman M.M., Sultana M. (2021). Microbial co-infections in COVID-19: Associated microbiota and underlying mechanisms of pathogenesis. Microb. Pathog..

[60] Taufik F.F., Natzir R., Patellongi I., Santoso A., Hatta M., Junita A.R., Syukri A., Primaguna M.R., Dwiyanti R., Febrianti A. (2022). In vivo and in vitro inhibition effect of propolis on Klebsiella pneumoniae: A review. Ann. Med. Surg..

[61] Almuhayawi M.S. (2020). Propolis as a novel antibacterial agent. Saudi J. Biol. Sci..

[62] Uyeki T.M., Hui D.S., Zambon M., Wentworth D.E., Monto A.S. (2022). Influenza. Lancet.

[63] WHO Influenza (Seasonal). https://www.who.int/news-room/fact-sheets/detail/influenza-(seasonal).

[64] Governa P., Cusi M.G., Borgonetti V., Sforcin J.M., Terrosi C., Baini G., Miraldi E., Biagi M. (2019). Beyond the biological effect of a chemically characterized poplar propolis: Antibacterial and antiviral activity and comparison with flurbiprofen in cytokines release by LPS stimulated human mononuclear cells. Biomedicines.

[65] Takemura T., Urushisaki T., Fukuoka M., Hosokawa-Muto J., Hata T., Okuda Y., Hori S., Tazawa S., Araki Y., Kuwata K. (2012). 3,4-dicaffeoylquinic acid, a major constituent of Brazilian propolis, increases TRAIL expression and extends the lifetimes of mice infected with the Influenza A Virus. Evid. Based Complement. Alternat. Med..

[66] Urushisaki T., Takemura T., Tazawa S., Fukuoka M., Hosokawa-Muto J., Araki Y., Kuwata K. (2011). Caffeoylquinic acids are major constituents with potent anti-influenza effects in brazilian green propolis water extract. Evid. Based Complement. Alternat. Med..

[67] Kujumgiev A., Tsvetkova I., Serkedjieva Y., Bankova V., Christov R., Popov S. (1999). Antibacterial, antifungal and antiviral activity of propolis of different geographic origin. J. Ethnopharmacol..

[68] Shimizu T., Hino A., Tsutsumi A., Park Y.K., Watanabe W., Kurokawa M. (2008). Anti-influenza virus activity of propolis in vitro and its efficacy against influenza infection in mice. Antivir. Chem. Chemother..

[69] Serkedjieva J., Manolova N., Bankova V. (1992). Anti-influenza virus effect of some propolis constituents and their analogues (esters of substituted cinnamic acids). J. Nat. Prod..

[70] Drago L., De Vecchi E., Nicola L., Gismondo M.R. (2007). In vitro antimicrobial activity of a novel propolis formulation (Actichelated propolis). J. Appl. Microbiol..

[71] Jin X., Ren J., Li R., Gao Y., Zhang H., Li J., Zhang J., Wang X., Wang G. (2021). Global burden of upper respiratory infections in 204 countries and territories, from 1990 to 2019. EClinicalMedicie.

[72] Esposito C., Garzarella E.U., Bocchino B., D'Avino M., Caruso G., Buonomo A.R., Sacchi R., Galeotti F., Tenore G.C., Zaccaria V. (2021). A standardized polyphenol mixture extracted from poplar-type propolis for remission of symptoms of uncomplicated upper respiratory tract infection (URTI): A monocentric, randomized, double-blind, placebo-controlled clinical trial. Phytomedicine.

[73] Di Pierro F., Zanvit A., Colombo M. (2016). Role of a proprietary propolis-based product on the wait-and-see approach in acute otitis media and in preventing evolution to tracheitis, bronchitis, or rhinosinusitis from nonstreptococcal pharyngitis. Int. J. Gen. Med..

[74] Cohen H.A., Varsano I., Kahan E., Sarrell E.M., Uziel Y. (2004). Effectiveness of an herbal preparation containing echinacea, propolis, and vitamin C in preventing respiratory tract infections in children: A randomized, double-blind, placebo-controlled, multicenter study. Arch. Pediatr. Adolesc. Med..

